# Health-Related Quality of Life and Frailty in Chronic Liver Diseases

**DOI:** 10.3390/life10050076

**Published:** 2020-05-24

**Authors:** Hiroki Nishikawa, Kazunori Yoh, Hirayuki Enomoto, Yoshinori Iwata, Yoshiyuki Sakai, Kyohei Kishino, Yoshihiro Shimono, Naoto Ikeda, Tomoyuki Takashima, Nobuhiro Aizawa, Ryo Takata, Kunihiro Hasegawa, Takashi Koriyama, Yukihisa Yuri, Takashi Nishimura, Shuhei Nishiguchi, Hiroko Iijima

**Affiliations:** 1 Department of Internal Medicine, Division of Gastroenterology and Hepatology, Hyogo College of Medicine, Nishinomiya, Hyogo 663-8501, Japan; mm2wintwin@ybb.ne.jp (K.Y.); enomoto@hyo-med.ac.jp (H.E.); yo-iwata@hyo-med.ac.jp (Y.I.); sakai429@hyo-med.ac.jp (Y.S.); hcm.kyohei@gmail.com (K.K.); yoshihiro19870729@yahoo.co.jp (Y.S.); nikeneko@hyo-med.ac.jp (N.I.); tomo0204@yahoo.co.jp (T.T.); nobu23hiro@yahoo.co.jp (N.A.); chano_chano_rt@yahoo.co.jp (R.T.); hiro.red1230@gmail.com (K.H.); takashi051114@yahoo.co.jp (T.K.); gyma27ijo04td@gmail.com (Y.Y.); tk-nishimura@hyo-med.ac.jp (T.N.); hiroko-i@hyo-med.ac.jp (H.I.); 2 Center for Clinical Research and Education, Hyogo College of Medicine, Nishinomiya, Hyogo 663-8501, Japan; 3 Kano General Hospital, Osaka 531-0041, Japan; nishiguchi@heartfull.or.jp

**Keywords:** chronic liver disease, health-related quality of life, SF-36, frailty

## Abstract

We sought to examine the relationship between frailty and health-related quality of life as evaluated using the 36-item Short-Form Health Survey (SF-36) questionnaire in Japanese chronic liver disease (CLD) patients (*n* = 341, 122 liver cirrhosis cases, median age = 66 years). Frailty was defined as a clinical syndrome in which three or more of the following criteria were met (frailty score 3, 4, or 5): unintentional body weight loss, self-reported exhaustion, muscle weakness (grip strength: <26 kg in men and <18 kg in women), slow walking speed (<1.0 m/s), and low physical activity. Robust (frailty score 0), prefrail (frailty score 1 or 2), and frailty were found in 108 (31.7%), 187 (54.8%), and 46 (13.5%) patients, respectively. In all eight scales of the SF-36 (physical functioning, role physical, bodily pain, general health perception, vitality, social functioning, role emotion, and mental health), and the physical component summary score and mental component summary score, each score was well stratified according to the frailty status (all *p* < 0.0001). In the multivariate analysis, age (*p* = 0.0126), physical functioning (*p* = 0.0005), and vitality (*p* = 0.0246) were independent predictors linked to the presence of frailty. In conclusion, Japanese CLD patients with frailty displayed poorer conditions, both physically and mentally.

## 1. Introduction

Health-related quality of life (Hr-QoL) is a globally used patient-reported clinical outcome for patients with various diseases and the number of pivotal clinical trials with Hr-QOL as additional study endpoints (most of them are secondary endpoints) has been increasing [1,2,3,4,5,6]. In particular, chronic liver diseases (CLDs) can significantly influence Hr-QOL [7,8,9,10,11,12,13]. Ameliorating Hr-QoL in patients with CLDs should be a pivotal treatment goal because of their unfavorable clinical and patient-reported outcomes and economic burdens [7,8,9,10,11,12,14,15]. The 36-item Short-Form Health Survey (SF-36) is one of the most extensively used modalities for assessing Hr-QoL [16,17,18,19,20].

Frailty is a condition in which physical and mental vitality (motor function, cognitive function, etc.) declines with age, living functions are impaired, and mental and/or physical fragility appears because of the coexistence of multiple chronic diseases [21,22,23]. With appropriate interventions, it is possible to maintain and improve living functions [21,22,23]. Frailty is a condition between a healthy condition and a nursing condition that requires support in daily life [21,22,23]. Recently, the concept of frailty has been enlarged to CLDs as clinical symptoms of impaired global physical function [14,24]. In patients with decreased liver function, frailty can occur regardless of age [25,26]. The median age of liver cirrhosis (LC) patients with frailty was reported to be 59 years [26]. Thus, disease-specific frailty can be a point of focus.

A previous meta-analysis reported that patients with frailty had significantly lower mental and physical QOL scores as assessed by the SF-36 than those with robust, community-dwelling older people [27]. In patients with end-stage liver disease referred for liver transplantation, a decreased Hr-QoL was negatively associated with frailty with statistical significance and not with the Model for End-Stage Liver Disease score [28]. Nixon et al. reported that frailty is independently associated with worse Hr-QoL in patients with severe chronic kidney injuries [29]. Frailty can thus be associated with Hr-QoL in various diseases. However, as far as we know, few data regarding the relevance between Hr-QoL and frailty in Japanese CLD patients are currently available. In this study, we sought to examine the relationship between frailty and Hr-QoL as evaluated by the SF-36 questionnaire in Japanese CLD patients.

## 2. Patients and Methods

### 2.1. Patients

A total of 341 CLD patients who visited our hospital between July 2015 and October 2019 were analyzed. All of these patients had data for frailty and SF-36. A case with CLD was a case confirmed to be accompanied by inflammation of the liver that had continued for 6 months or more at the time of the visit or in the past. LC was determined via histological findings, imaging studies, and/or laboratory data. Frailty was defined as a clinical syndrome in which three or more of the following criteria were met (i.e., frailty score 3, 4, or 5): unintentional body weight (BW) loss (2 kg, 3 kg, or more BW loss within the past 6 months), self-reported exhaustion, muscle weakness (grip strength (GS): < 26 kg in men and < 18 kg in women), slow walking speed (WS, < 1.0 m/s), and low physical activity (doing light exercise or not), while prefrail was defined as patients with one or two above-mentioned phenotypes (i.e., frailty score 1 or 2). Patients with none of five phenotypes were regarded as having a robust status (frailty score 0) [30,31]. These criteria are reported by Satake and Arai as the Japanese version of Cardiovascular Health Study (CHS) criteria [30]. GS was measured according to the current guidelines [32]. In all analyzed subjects, a six-meter walking test was done. The six-meter walking test was done twice in all subjects and the WS (m/s) was defined as the mean value of them. The assessment of frailty and the questionnaire using SF-36 in each patient were done on the same day. Patients with large ascites or overt hepatic encephalopathy who are potentially involved in frailty were excluded due to unreliable self-reporting.

### 2.2. Questionnaire

Our patients were requested to fill out the Japanese version of the SF-36 (self-reported questionnaire). The SF-36 consists of 36 items that are classified into multiple scales (a total of eight scales): physical functioning, role physical, bodily pain, general health perception, vitality, social functioning, role emotion, and mental health [33,34,35,36]. Each scale is scored from 0 to 100 points, and a higher score on the scale indicates a better health status [33,34,35,36]. The physical component summary score (PCS) and the mental component summary score (MCS) were additionally calculated and examined.

### 2.3. Our Study

We retrospectively examined the relationship between the frailty status and the values of the eight scales of SF-36, the PCS, and the MCS. Ethical approval was obtained from the ethics committee of our hospital. The protocol in the study rigorously observed all regulations of the Declaration of Helsinki.

### 2.4. Statistical Considerations

JMP 14 software (SAS Institute Inc., Cary, NC, USA) was used to perform the statistical analysis. For the numerical variables, Student’s *t*-test, the Mann–Whitney U-test, analysis of variance, or the Kruskal–Wallis test was used to assess group characteristics when appropriate. Numerical data were expressed as the median value (interquartile range (IQR)). Baseline significant items in our univariate analysis were subject to the multivariate logistic regression analysis to select candidate parameters. The statistical significance level was set at *p* < 0.05.

## 3. Results

### 3.1. Patient Baseline Data

**Figure 1:**
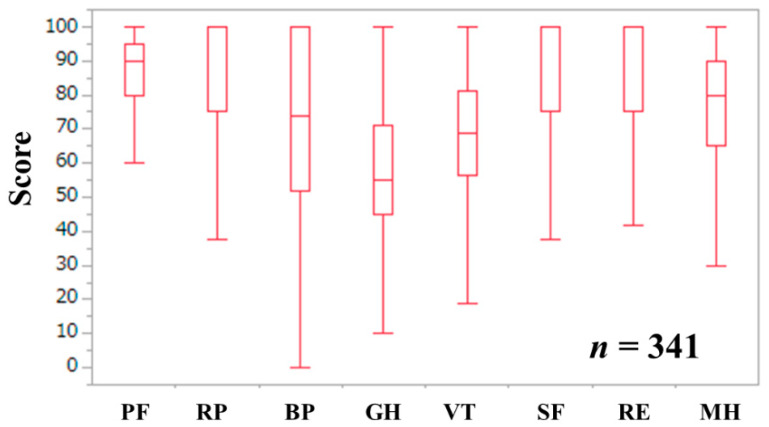
Scores for the eight scales in the SF-36 for all cases (*n* = 341). PF indicates physical functioning, RP indicates role physical, BP indicates bodily pain, GH indicates general health perception, VT indicates vitality, SF indicates social functioning, RE indicates role emotion, and MH indicates mental health.

The baseline data in this study (*n* = 341, 164 men and 177 women, median age = 66.0 years) are indicated in Table 1. LC was identified at baseline in 122 cases (35.8%). Of these, there were 85 patients with Child–Pugh A, 33 with Child–Pugh B, and 4 with Child–Pugh C. ALBI (albumin-bilirubin) grade 1 was found in 256 patients (75.1%), grade 2 in 78 (22.9%), and grade 3 in 7 (2.1%). The ALBI grade means a grading system with a combined serum albumin level and bilirubin level in patients with liver diseases; it has been introduced as a prognostic system alternative to the Child–Pugh classification system [37]. Non-B, non-C related CLDs included: alcoholic liver injury in 6, autoimmune liver disease in 32; fatty liver or non-alcoholic steatohepatitis in 17; and others, including unknown etiology after sufficient screening, in 44. The median (IQR) WS was 1.30 (1.10, 1.44) m/s. Fifty-one patients (15.0%) displayed a WS decrease (i.e., <1.0 m/s). One-hundred-and-sixty-eight patients (49.3%) reported exhaustion. Fifteen patients (4.4%) reported BW loss. Ninety patients (26.4%) reported low physical activity. The frailty score ranged from 0 to 5 (median, 1). Robust (frailty score 0), prefrail (frailty score 1 or 2), and frailty (frailty score 3 or more) were identified in 108 (31.7%), 187 (54.8%), and 46 (13.5%) patients, respectively. The median (IQR) age in patients with robust, prefrail, and frailty for all cases were 61 (50.25, 68) years, 66 (56, 73) years, and 73 (68, 75.25) years (overall *p*-value < 0.0001). The median (IQR) scores of the eight scales of SF-36 were: 90 (80, 95) in physical function, 100 (75, 100) in role physical, 74 (52, 100) in bodily pain, 55 (45, 71) in general health perception, 68.8 (56.2, 81.3) in vitality, 100 (75, 100) in social functioning, 100 (75, 100) in role emotion, and 80 (65, 90) in mental health (Figure 1). The median (IQR) PCS and MCS were 51.1 (41.2, 54.3) and 53.6 (46.0, 59.2).

### 3.2. Scores of the Eight Scales of the SF-36, the PCS, and the MCS Relative to the Frailty Status

**Figure 2:**
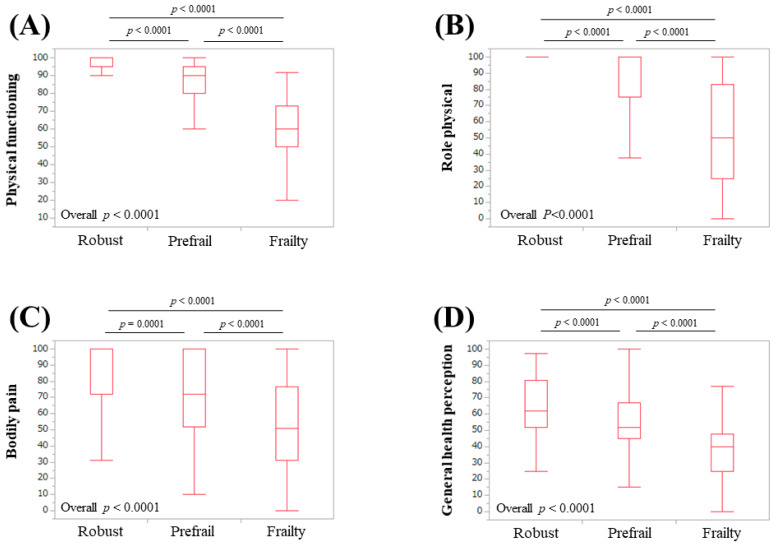
Scores for four of the scales of the SF-36 relative to the frailty status for all cases: (**A**) physical functioning, (**B**) role physical, (**C**) bodily pain, and (**D**) general health perception.

**Figure 3:**
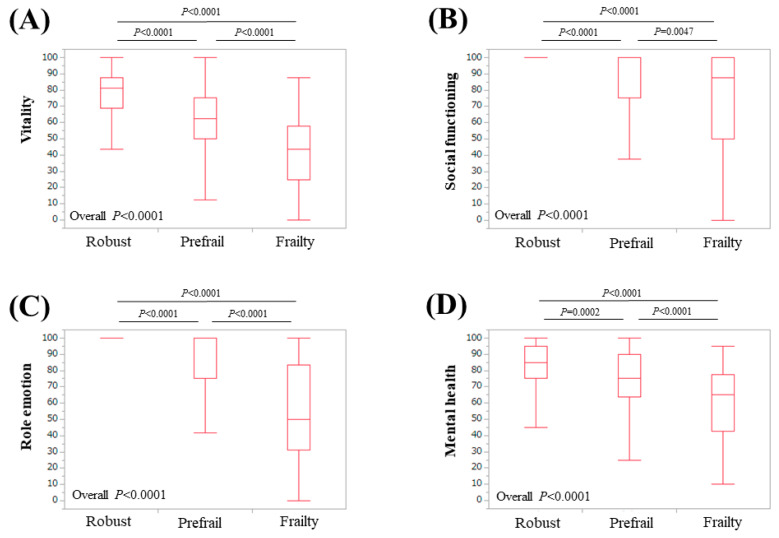
Scores for four of the scales of the SF-36 relative to the frailty status for all cases: (**A**) vitality, (**B**) social functioning, (**C**) role emotion, and (**D**) mental health.

**Figure 4:**
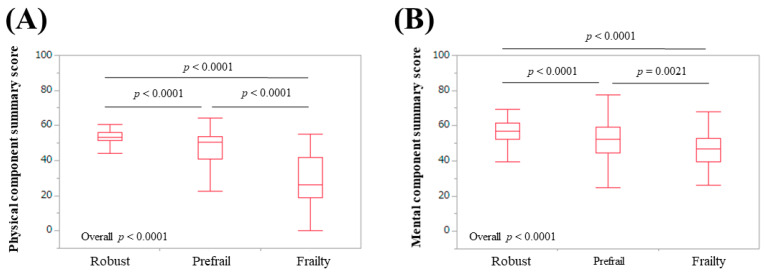
Physical component summary score (**A**) and mental component summary score (**B**) relative to the frailty status for all cases.

The median (IQR) physical functioning scores in patients classified as robust, prefrail, and frail were 95 (95,100), 90 (80, 95), and 60 (50, 72.9), respectively (*p*-values: robust vs. prefrail, *p* < 0.0001; prefrail vs. frail, *p* < 0.0001; frail vs. robust, *p* < 0.0001; overall *p* < 0.0001) (Figure 2A). The median (IQR) role physical scores in patients classified as robust, prefrail, and frail were 100 (100, 100), 100 (75, 100), and 50 (25, 82.85), respectively (*p*-values: robust vs. prefrail, *p* < 0.0001; prefrail vs. frail, *p* < 0.0001; frail vs. robust, *p* < 0.0001; overall *p* < 0.0001) (Figure 2B). The median (IQR) bodily pain scores in patients classified as robust, prefrail, and frail were 100 (72, 100), 72 (52, 100), and 51 (31, 76.5), respectively (*p*-values: robust vs. prefrail, *p* = 0.0001; prefrail vs. frail, *p* < 0.0001; frail vs. robust, *p* < 0.0001; overall *p* < 0.0001) (Figure 2C). The median (IQR) general health perception scores in patients classified as robust, prefrail, and frail were 62 (52, 80.75), 52 (45, 67), and 40 (25, 47.75), respectively (*p*-values: robust vs. prefrail, *p* < 0.0001; prefrail vs. frail, *p* < 0.0001; frail vs. robust, *p* < 0.0001; overall *p* < 0.0001) (Figure 2D). The median (IQR) vitality scores in patients classified as robust, prefrail, and frail were 81.3 (68.8, 87.5), 62.5 (50, 75), and 43.8 (25, 57.85), respectively (*p*-values: robust vs. prefrail, *p* < 0.0001; prefrail vs. frail, *p* < 0.0001; frail vs. robust, *p* < 0.0001; overall *p* < 0.0001) (Figure 3A). The median (IQR) social functioning scores in patients classified as robust, prefrail, and frail were 100 (100, 100), 100 (75, 100), and 87.5 (50, 100), respectively (*p*-values: robust vs. prefrail, *p* < 0.0001; prefrail vs. frail, *p* = 0.0047; frail vs. robust, *p* < 0.0001; overall *p* < 0.0001) (Figure 3B). The median (IQR) role emotion scores in patients classified as robust, prefrail, and frail were 100 (100, 100), 100 (75, 100), and 50 (31.225, 83.3), respectively (*p*-values: robust vs. prefrail, *p* < 0.0001; prefrail vs. frail, *p* < 0.0001; frail vs. robust, *p* < 0.0001; overall *p* < 0.0001) (Figure 3C). The median (IQR) mental health scores in patients classified as robust, prefrail, and frail were 85 (75, 95), 75 (60, 90), and 62.5 (43.75, 76.25), respectively (*p*-values: robust vs. prefrail, *p* = 0.0002; prefrail vs. frail, *p* < 0.0001; frail vs. robust, *p* < 0.0001; overall *p* < 0.0001) (Figure 3D). The median (IQR) PCS in patients classified as robust, prefrail, and frail were 53.3 (51.2, 56), 50.5 (41.05, 53.53), and 26.3 (19.1, 42), respectively (*p*-values: robust vs. prefrail, *p* < 0.0001; prefrail vs. frail, *p* < 0.0001; frail vs. robust, *p* < 0.0001; overall *p* < 0.0001) (Figure 4A). The median (IQR) MCS in patients classified as robust, prefrail, and frail were 56.7 (52.2, 61.3), 52.25 (44.48, 59), and 46.7 (39.7, 52.8), respectively (*p*-values: robust vs. prefrail, *p* < 0.0001; prefrail vs. frail, *p* = 0.0021; frail vs. robust, *p* < 0.0001; overall *p* < 0.0001) (Figure 4B).

### 3.3. Subgroup Analysis 1: Scores of the Eight Scales of the SF-36 Relative to the Frailty Status in LC Patients

**Figure 5:**
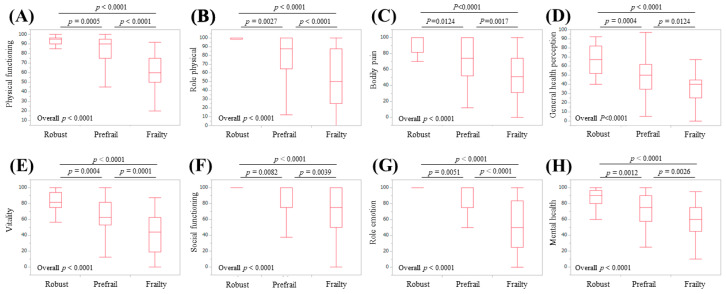
Scores for the eight scales of the SF-36 relative to the frailty status for LC cases (*n* = 122): (**A**) physical functioning, (**B**) role physical, (**C**) bodily pain, (**D**) general health perception, (**E**) vitality, (**F**) social functioning, (**G**) role emotion, and (**H**) mental health.

In LC patients (*n* = 122), robust, prefrail, and frailty statuses were identified in 22 (18.0%), 69 (56.6%), and 31 (25.4%) patients, respectively. The median (IQR) ages in LC patients classified as robust, prefrail, and frail in LC patients were 67.5 (57, 72.25) years, 68 (61.5, 73) years, and 73 (66, 76) years, respectively (overall *p*-value = 0.6884). For all eight scales, the overall *p*-values among groups of robust, prefrail, and frail patients reached significance (overall *p*-values: *p* < 0.0001 for all scales) (Figure 5).

### 3.4. Subgroup Analysis 2: Scores of the Eight Scales of the SF-36 Relative to the Frailty Status in Non-LC Patients

**Figure 6:**
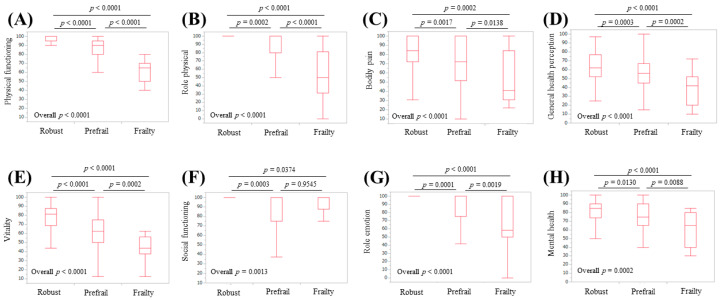
Scores for the eight scales of the SF-36 relative to the frailty status for non-LC cases (*n* = 219): (**A**) physical functioning, (**B**) role physical, (**C**) bodily pain, (**D**) general health perception, (**E**) vitality, (**F**) social functioning, (**G**) role emotion, and (**H**) mental health.

In non-LC patients (*n* = 219), robust, prefrail, and frail statuses were identified in 86 (39.3%), 118 (53.9%), and 15 (6.8%) patients, respectively. The median (IQR) ages in non-LC patients classified as robust, prefrail, and frail were 59.5 (48, 66) years, 64.5 (52, 72) years, and 73 (70, 75) years (overall *p*-value < 0.0001). For all eight scales, the overall *p*-values of the robust, prefrail, and frail groups reached significance (overall *p*-values: *p* = 0.0013 for social functioning, *p* = 0.0002 for mental health, *p* < 0.0001 for the remaining six scales) (Figure 6).

### 3.5. Subgroup Analysis 3: Scores of the Eight Scales of the SF-36 Relative to the Frailty Status in Male Patients

**Figure 7:**
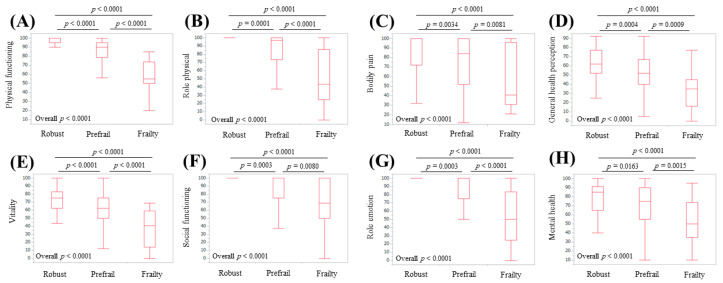
Scores for the eight scales of the SF-36 relative to the frailty status for male cases (*n* = 164): (**A**) physical functioning, (**B**) role physical, (**C**) bodily pain, (**D**) general health perception, (**E**) vitality, (**F**) social functioning (**G**) role emotion, and (**H**) mental health.

In male patients (*n* = 164), robust, prefrail, and frail statuses were identified in 54 (32.9%), 90 (54.9%), and 20 (12.2%) patients, respectively. For all eight scales, the overall *p*-values of the robust, prefrail, and frail groups reached significance (overall *p*-values: *p* < 0.0001 in all scales) (Figure 7).

### 3.6. Subgroup Analysis 4: Scores of the Eight Scales of the SF-36 Relative to the Frailty Status in Female Patients

**Figure 8:**
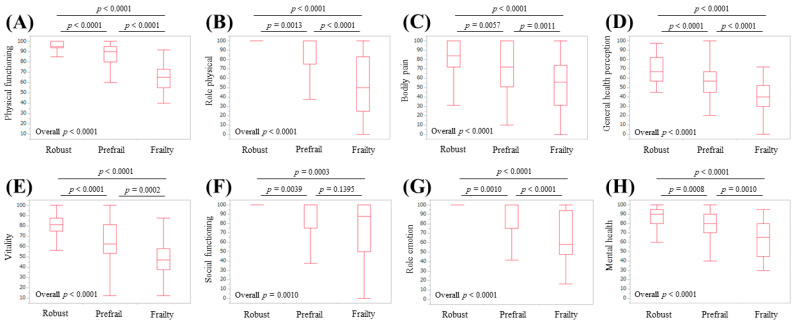
Scores for the eight scales of the SF-36 relative to the frailty status for female cases (*n* = 177): (**A**) physical functioning, (**B**) role physical, (**C**) bodily pain, (**D**) general health perception, (**E**) vitality, (**F**) social functioning, (**G**) role emotion, and (**H**) mental health.

In female patients (*n* = 177), robust, prefrail, and frail statuses were identified in 54 (30.5%), 97 (54.8%), and 26 (14.7%) patients, respectively. For all eight scales, the overall *p*-values of the robust, prefrail, and frail groups reached significance (overall *p*-values: *p* = 0.0010 for social functioning and *p* < 0.0001 for the remaining seven scales) (Figure 8).

### 3.7. Univariate and Multivariate Analysis of Factors Linked to Frailty

A univariate analysis identified the following significant factors linked to frailty; age (*p* = 0.0002), presence of LC (*p* < 0.0001), serum albumin (*p* < 0.0001), physical functioning (*p* < 0.0001), role physical (*p* < 0.0001), bodily pain (*p* < 0.0001), general health perception (*p* < 0.0001), vitality (*p* < 0.0001), social functioning (*p* < 0.0001), role emotion (*p* < 0.0001), and mental health (*p* < 0.0001) (Table 2). In the multivariate analysis, age (*p* = 0.0126), physical functioning (*p* = 0.0005), and vitality (*p* = 0.0246) were independent predictors linked to frailty, while the presence of LC tended to be significant (*p* = 0.0685) (Table 2).

## 4. Discussion

Frailty is a multi-dimensional disease concept that represents the end-stage manifestation of disorders in numerous physiological systems, resulting in physiological reserve decline and an increase in vulnerability to health stressors [15]. The SF-36 can measure the Hr-QoL for various diseases and can compare the Hr-QoL between patients with different diseases [33,34,35,36]. To the best of our knowledge, this is the first study elucidating the relationship between frailty and Hr-QoL as assessed using the SF-36 in Japanese patients with CLDs. In our data, the lowest median score of general health (median value = 55) among the eight scales may be due to the long-standing disease burden of CLDs. Meanwhile, scores of role physical (median value = 100), social functioning (median value = 100), and role emotion (median value = 100) were well maintained.

Physical functioning at the top of the eight scales in the SF-36 is most strongly associated with physical health. Mental health at the bottom of the eight scales in the SF-36 is most strongly associated with mental health [33,34,35,36]. For the six scales between physical functioning and mental health, the higher the scale, the stronger the relationship with physical health, and the lower the scale, the stronger the relationship with mental health [33,34,35,36]. This structure helped us interpret our results. In our results, the scores of the eight scales of the SF-36, the PCS, and the MCS were all well stratified according to the frailty condition. In our multivariate analysis for frailty, physical functioning and vitality were independent predictors linked to frailty. Physical functioning, role physical, bodily pain, and general health perception are categorized as physical health, while vitality, social functioning, role emotion, and mental health are categorized as mental health [33,34,35,36]. Taken together, frailty in CLDs could be associated with both physical aspects and mental aspects. Before the current analysis, we hypothesized that frailty in CLDs as assessed by the Japanese version of the CHS criteria was linked to only physical health because five phenotypes for the assessment of frailty (unintentional BW loss, self-reported exhaustion, muscle weakness, slow WS, and low physical activity) are phenotypes mainly about physical health. The CHS criteria are a representative assessment tool for physical frailty [30,31]. For that reason, the current results were surprising to us. Role emotion was significantly stratified with a strong *p*-value (all, *p* < 0.0001) relative to the frailty status for all analyses. Role emotion indicates the effect of social activities on the psychological state [33,34,35,36]. Social frailty is clearly defined as poor participation in social networks and the awareness of lacking in contacts and surrounding support [38]. The CHS criteria may be somewhat linked to social frailty.

In our 46 frail patients, 34 patients (73.9%) had a GS decline. GS decline, rather than muscle mass decline, seems to be closely associated with poor QOL in CLDs [39]. Sarcopenia as assessed in terms of muscle mass decline and muscle strength decline or low physical activity is a key component in physical frailty, which is in line with our current data [15]. The prevalence of frailty may be difficult to report precisely because several assessment tools and cut-off values are currently available [15]. Lai et al. demonstrated that out of 983 LC patients, 151 (15%) displayed frailty and the median age of frail LC patients was 59 years [26]. In our data, 46 (13.5%) were identified as frail, and in patients less than 65 years (*n* = 155), 80 patients (51.6%) were prefrail and 8 patients (5.2%) were frail. We believe that frailty in CLDs should not be restricted to elderly patients and a disease-specific frailty condition should be fully assessed, even in younger CLD patients. Muscle protein synthesis may decrease in younger advanced CLD patients due to protein energy malnutrition or other metabolic disorders [15,40]. Out of our eight frail patients less than 65 years old, 7 (87.5%) had LC. In this sense, disease-specific frailty should be emphasized. In our multivariate analysis for frailty, age was also an independent factor, along with physical functioning and vitality, while the presence of LC tended to be significant. In LC patients, age did not significantly influence the frailty status (*p* = 0.6884). Inversely, in non-LC patients, age significantly influenced the frailty status (*p* < 0.0001). Considering these results, frailty in CLDs may be involved in both aging-related factors and liver-function-related factors.

Several limitations of this study need to be acknowledged. First, this study was a single-center observational study with a retrospective nature. Second, the study data was derived from Japanese CLD population data; additional exams on other ethnic backgrounds are needed to further verify and extend the application to these ethnic backgrounds. Third, GS or WS (i.e., one of phenotypes for frailty assessment) can vary depending on measurement conditions. Fourth, patients with large ascites or overt hepatic encephalopathy who are potentially frail were excluded due to unreliable self-reporting, creating bias. Our data should be therefore interpreted with caution. Nevertheless, our study results denoted that frailty in Japanese CLD patients had decreased Hr-QOL, as evaluated by the SF-36 in terms of both physical and mental components. In conclusion, Japanese CLD patients with frailty display poorer conditions, both physically and mentally. Appropriate interventions will be required for such patients.

## Figures and Tables

**Table 1:** Baseline characteristics (*n* = 341).

Variables	All Cases (*n* = 341)
Age (years)	66 (55, 72)
Gender, male/female	164/177
Liver disease etiology HCV-related/HBV-related/HBV- and HCV-related/NBNC-related	174/61/7/99
Presence of frailty, yes/no	46/295
Presence of LC, yes/no	122/219
Body mass index (kg/m^2^)	22.7 (20.5, 25.65)
Walking speed (m/s)	1.303 (1.1005, 1.4445)
Grip strength (kg), male	33.3 (27.925, 38.925)
Grip strength (kg), female	20.8 (17.6, 24.45)
Total bilirubin (mg/dL)	0.8 (0.6, 1.1)
Serum albumin (g/dL)	4.3 (4.0, 4.5)
ALBI score	−2.9 (−3.12, −2.6)
ALBI grade, 1/2/3	256/78/7
Prothrombin time (%)	91.2 (80.55, 99.05)
Platelet count (×10^4^/mm^3^)	17.5 (12.6, 22.0)
AST (IU/L)	25 (19, 34)
ALT (IU/L)	19 (14, 33)
Total cholesterol (mg/dL)	181 (151.25, 213)
HbA1c (NGSP)	5.7 (5.4, 6.1)
Scales of SF-36	
Physical functioning	90 (80, 95)
Role physical	100 (75, 100)
Bodily pain	74 (52, 100)
General health perception	55 (45, 71)
Vitality	68.8 (56.2, 81.3)
Social functioning	100 (75, 100)
Role emotion	100 (75, 100)
Mental health	80 (65, 90)
Physical component summary score	51.1 (41.2, 54.3)
Mental component summary score	53.6 (46.0, 59.2)

Data are expressed as the number or median value (interquartile range). HCV: hepatitis C virus, HBV: hepatitis B virus, NBNC: non-B and non-C, LC: liver cirrhosis, ALBI: albumin-bilirubin, AST: aspartate aminotransferase, ALT: alanine aminotransferase, NGSP: National Glycohemoglobin Standardization Program, SF-36: 36-item Short-Form Health Survey.

**Table 2:** Univariate and multivariate analyses of factors linked to frailty.

Variables	Univariate Analysis *p*-Value	Multivariate Analysis		
		Estimates	SE	*p*-Value
Age (years)	0.0002	−0.0499	0.020	0.0126
Gender	0.5292			
Presence of LC	<0.0001	−0.484	0.265	0.0685
Body mass index	0.3701			
Total bilirubin	0.9042			
Serum albumin	<0.0001	0.259	0.413	0.5308
Prothrombin time	0.2037			
Platelet count	0.0543			
AST	0.0992			
ALT	0.6717			
Total cholesterol	0.0815			
HbA1c (NGSP)	0.1741			
Physical functioning	<0.0001	0.0434	0.0125	0.0005
Role physical	<0.0001	0.00769	0.0102	0.4531
Bodily pain	<0.0001	−0.000804	0.0095	0.9327
General health perception	<0.0001	0.00357	0.0139	0.7976
Vitality	<0.0001	0.0349	0.0155	0.0246
Social functioning	<0.0001	−0.0169	0.0117	0.1481
Role emotion	<0.0001	0.0158	0.0106	0.1384
Mental health	<0.0001	−0.00394	0.0146	0.7877

SE: standard error, LC: liver cirrhosis, AST: aspartate aminotransferase, ALT: alanine aminotransferase, NGSP: National Glycohemoglobin Standardization Program.

## Abbreviation

Hr-QOL	health-related quality of life
CLDs	chronic liver diseases
SF-36	36-item Short-Form Health Survey
LC	liver cirrhosis
BW	body weight
GS	grip strength
WS	walking speed
CHS	Cardiovascular Health Study
PCS	physical component summary score
MCS	mental component summary score
IQR	interquartile range
ALBI	albumin-bilirubin
